# Unlocking Efficient Ultrafast Bound-Electron Optical Nonlinearities via Mirror Induced Quasi Bound States in the Continuum

**DOI:** 10.1021/acs.nanolett.3c04431

**Published:** 2024-01-23

**Authors:** Guoce Yang, Monica S. Allen, Jeffery W. Allen, Hayk Harutyunyan

**Affiliations:** † Department of Physics, Emory University, Atlanta, Georgia 30322, United States; ‡ Air Force Research Laboratory, Munitions Directorate, Eglin AFB, Florida 32542, United States

**Keywords:** optical switching, bound states in the continuum, metasurfaces, nonlinear refractive index, two photon transition

## Abstract

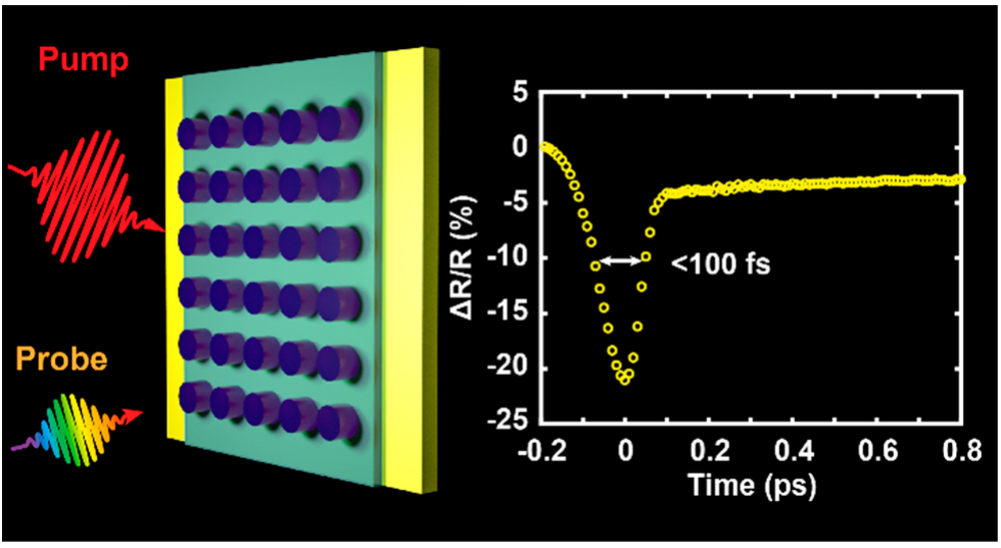

The operation of photonic devices often relies on modulation of their refractive index. While the sub-bandgap index change through bound-electron optical nonlinearity offers a faster response than utilizing free carriers with an overbandgap pump, optical switching often suffers from inefficiency. Here, we use a recently observed metasurface based on mirror-induced optical bound states in the continuum, to enable superior modulation characteristics. We achieve a pulsewidth-limited switching time of 100 fs, reflectance change of 22%, remarkably low energy consumption of 255 μJ/cm^2^, and an enhancement of modulation contrast by a factor of 440 compared to unpatterned silicon. Additionally, the narrow photonic resonance facilitates the detection of the dispersive nondegenerate two-photon nonlinearity, allowing tunable pump and probe excitation. These findings are explained by a two-band theoretical model for the dispersive nonlinear index. The demonstrated efficient and rapid switching holds immense potential for applications, including quantum photonics, sensing, and metrology.

Time-varying metasurfaces are powerful reconfigurable platforms to realize novel and enhanced active photonic functionalities through subwavelength control of amplitude, phase, and polarization of light.^1−7^ Promising applications of such highly controllable light–matter interactions include ultrafast all-optical switching, modulation, and beam steering.^8−13^ Such platforms and devices are enabled by a class of optical nonlinearities based on the intensity-dependent refractive index change.^14^ These types of ultrafast optical phenomena are classified based on the physical processes harnessed for index modulation. One approach is based on the thermal optical effect, where the refractive index is tuned as a function of temperature. The energy of light is absorbed by materials and converted to heat, which induces a temperature increase in the lattice, changing its refractive index. The switching time depends on the thermal exchange dynamics, which can be accelerated to tens of ps by miniaturizing the device footprint.^15^ Another approach is based on generating free carriers through the interband transitions by pumping materials with photons that have energies above the bandgap. Deep signal modulation is observed with relatively low power consumption, and the free carrier relaxation time is limited by the electron–hole recombination rate with time scales ranging from subps to ps with direct bandgap and amorphous materials used to boost the process.^16,17^ A third method to modulate intensity-dependent refractive index is based on tuning the effective mass of free carriers occupying the nonparabolic conduction band via the intraband transitions pumped by sub-bandgap energy photons.^18−20^ The relaxation time can be shortened to hundreds of femtoseconds since electron–electron and electron–phonon interactions dominate the relaxation process. However, the tuning range of effective mass is relatively small, and it is necessary to work in the epsilon-near-zero (ENZ) range to observe a significant modulation^21−24^ which requires accurate carrier concentration control and engineering the defects in materials, complicating sample fabrication.^25,26^

The interaction of light with bound electrons can be an alternative to free carrier effects, inducing a third-order optical nonlinearity, called the optical Kerr effect, with an ultrashort response time of <1 fs.^27^ However, this process is inefficient and results in weak optical modulation contrast even in highly nonlinear materials such as silicon, making it impractical in applications. Optical nanoresonators can be used to amplify the modulation strength by leveraging the sensitivity of resonances to small changes in the refractive index. For example, the magnetic and anapole resonant modes of high refractive index nanodisk have been used to enhance ultrafast optical signals.^5,28,29^ However, the relatively low-quality factors (Q factors) of these modes limit the switching efficiency. Moreover, the nonlinear refractive index in these systems has always been estimated using a dispersion-less constant^29,30^ that is a good approximation when both pump and probe photon energies are far away from the electronic transition energy. When this is not the case and the sum of the pump and probe photon energies is near the transition energy, the nonlinearity becomes strongly dispersive and nondegenerate for different pump and probe wavelengths.^31−33^ It is necessary to account for this dispersion, because it leads to a different ultrafast optical response depending on the intrinsic ground-state optical resonance of the sample and the external pump wavelength. This aspect has never been explored in ultrafast spectroscopy studies and can provide another degree of freedom for the design of ultrafast, time-varying devices and metamaterials.

This work demonstrates a novel and efficient pulsewidth-limited ultrafast all-optical switching platform using an amorphous silicon metasurface. Subwavelength Si nanodisks fabricated on top of a gold mirror form the basis of the metasurface that supports photonic bound states in the continuum (BICs).^34−36^ The BIC modes are created by the coupling of electric dipole (ED) or magnetic dipole (MD) resonances to their mirror image. Unlike most designs achieving BIC resonances in photonic metasurfaces, our approach does not require breaking the spatial symmetry making it more attractive to simplify design space.^37^ We show an ultrafast response time of ∼100 fs, limited by the instrument response function (IRF) of the experimental setup. The efficiency of the BIC platform is shown by implementing a relative reflection change of 22% at a low pump fluence of 255 μJ/cm^2^, corresponding to only 0.9 pJ per nanodisk. To the best of our knowledge, this is the lowest power consumption rate demonstrated for large-amplitude, pulse-width-limited optical switching. We also show that the transient spectral shape can be tuned by both the BIC resonance and the pump wavelength due to the nondegenerate two-photon absorption (TPA) involving one pump and one probe photon. We develop a theoretical model based on a two-band transition model and the Kramers–Kronig (K–K) relationship to simulate the ultrafast transient spectra and the dispersive complex nonlinear refractive index, which agrees well with the experimental data.

Figure 1a shows a three-dimensional (3D) schematic and a scanning electron microscope (SEM) image of the metasurface. The height of the silicon disks is 305 nm, and the periodicity is twice the disk diameter for all samples. The array size of the metasurface is 100 × 100 μm^2^. A spacer layer of SiO_2_ separates the Si disks and the gold surface with a thickness of 94 nm, which plays a fundamental role in forming BICs by controlling the coupling of the resonance to its image by the propagation phase difference between them.^37^ The high-index nanodisk resonators support various Mie resonances, including both ED and MD resonances. Ideal BICs are obtained by making the phase difference equal to either an odd or even multiple of π, corresponding to the antiparallel state [1, −1]^T^ and the parallel state [1, 1]^T^. Considering that ED always oscillates out of phase with its mirror dipole, whereas MD is in phase, these modes can form BIC resonances. The simulated mode profiles of the two types of BICs are shown in Figure 1b and 1c, respectively. The orientation distribution of the electric field vector inside the Si disk indicates the characteristics of ED and MD modes. We calculate the propagation phase using the refractive index of Si and SiO_2_, and it is estimated to be 3π/2 for ED BIC resonance from the center of the disks to the gold surface. Correspondingly, the estimated phase difference is π for the MD BIC case. The existence of phase related BICs is confirmed by simulating reflectance spectra with the fixed disk diameter of 320 nm and varying thickness of the SiO_2_, shown in Figure 1d. BIC turning points occur for both the ED and MD resonant branches on the spectral map. The Q factor of the quasi-BIC modes increases as we approach the inflection point. There are two perfect absorption points (zero reflectance) on each branch, which can be explained by the system’s evolution between overcoupling and undercoupling states according to temporal coupled mode theory. From a topological perspective, the perfect absorption points emerge in pairs linked to the phase singularity points.^38,39^ The phase difference can be tuned by changing either the thickness of the SiO_2_ layer or the diameter of the nanodisks. Controlling the thickness of the SiO_2_ spacer is difficult, and therefore, we opt to vary the disk diameter where the phase difference is tuned by changing the effective wavenumber *k = n*_eff_ω_0_/*c*, where *n*_eff_ is the effective refractive index of the medium between the center of disk and gold surface and *c* is the light speed in vacuum. Simulations of diameter-dependent reflectance spectra are shown in Figure 1e and the corresponding measured reflectance spectra are shown in Figure 1f and have excellent agreement with each other. Several selected spectral curves and retrieved Q factors are shown in the Supporting Information (Figure S1). A BIC turning point is found in the MD branch but does not appear in the ED counterpart because the thin SiO_2_ spacer results in significant near-field coupling between the ED mode and surface plasmon polaritons (SPPs) that can be ignored when the spacer is thicker than 200 nm. Additional simulations with varying thicknesses of the spacers and the comparison with the Si metasurface without the mirror are shown in Supporting Information (Figure S2). A similar comparison of reflectance spectra with varying diameters but a fixed thickness of the spacer is also shown in Supporting Information (Figure S3). Analysis of the ground-state spectra reveals that the line width of the ED and MD resonances of the nanodisks is significantly reduced by the BICs.

**Figure 1:**
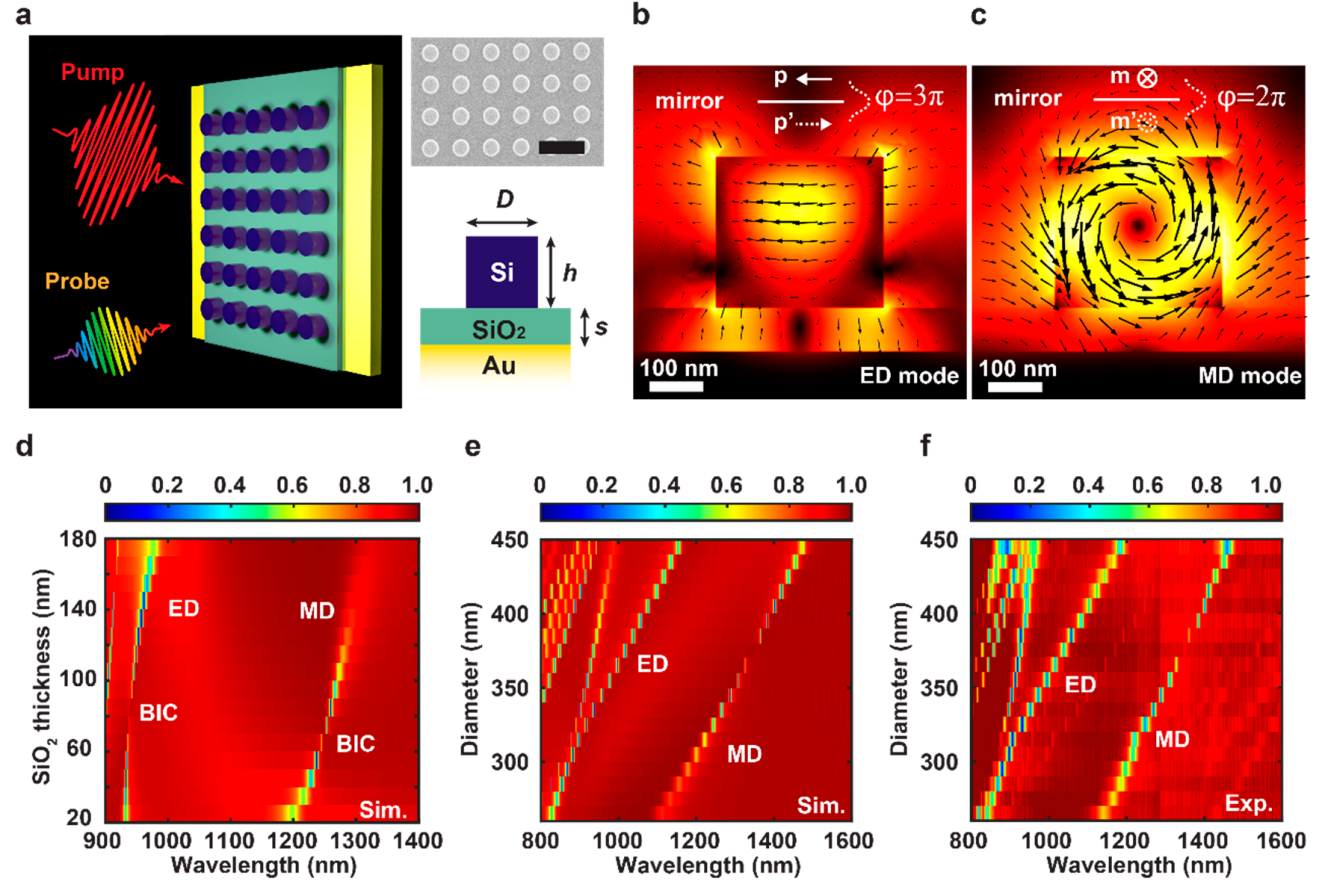
BICs enabled by ED and MD resonances coupled to a mirror. (a) Schematic and SEM images of the BIC metasurface. Scale bar: 1 μm. (b, c) Simulated electric field profile of the ED mode and the MD mode on the cross section of a unit cell. The insets show the phase mechanism of the ED and MD BIC modes. (d) Simulated reflectance spectra for varying thicknesses of SiO_2_ showing the spectral evolution of the ED and MD resonances and the formation of the BIC points. (e) Simulated reflectance spectra for varying Si nanodisk diameters. (f) Experimentally measured reflectance spectra with the normal incidence of the fabricated metasurfaces with different diameters of Si nanodisks.

Next, we exploit these high Q quasi-BIC MD resonances to enhance the modulation contrast of all-optical switching using the pump–probe measurement setup shown in Supporting Information (Figure S4). The pump wavelength is detuned from the resonances; therefore the optical absorption-induced nonlinearity in gold can be ignored. Here, the optical switching can be achieved by spectrally tuning the high-Q resonances which are sensitive to the pump-induced ultrafast change of the silicon nanodisks’ refractive index. It should be noted that the incident angle of the pump and the probe beam is 30° and 5°, respectively. Such a configuration does not significantly affect the pump electric field in Si pillars and the ground-state resonance of the pillars (see Supporting Information, Figures S5 and S6). The representative pump–probe transient spectra of reflectance change Δ*R*/*R* as a function of the probe wavelength are shown in Figure 2a. The examination of the time evolution of the spectra reveals two kinetic processes. An ultrafast ∼100 fs response dominates at the time zero followed by a relatively slow process with a decay time of ∼1 ps. The transient spectra feature enhanced Δ*R*/*R* for both high-Q ED and MD quasi-BIC resonant modes. Δ*R*/*R* values at the MD resonance for various diameters and pump wavelengths are shown in Figure 2b. The theoretical maximum for the measured |Δ*R*/*R*| should occur at the perfect absorption point (*D* = 340 nm); however, this is not the case in the experimentally observed response which peaks at *D* = 300 nm. The reason for this disagreement is the limited spectral resolution of the pump–probe setup. The narrower and deeper resonance can enhance the ultrafast modulation contrast; however, the significantly decreased resonant bandwidth (∼7.5 nm at *D* = 340 nm) approaches the spectral resolution (∼3.5 nm), which reduces the measured contrast.

**Figure 2:**
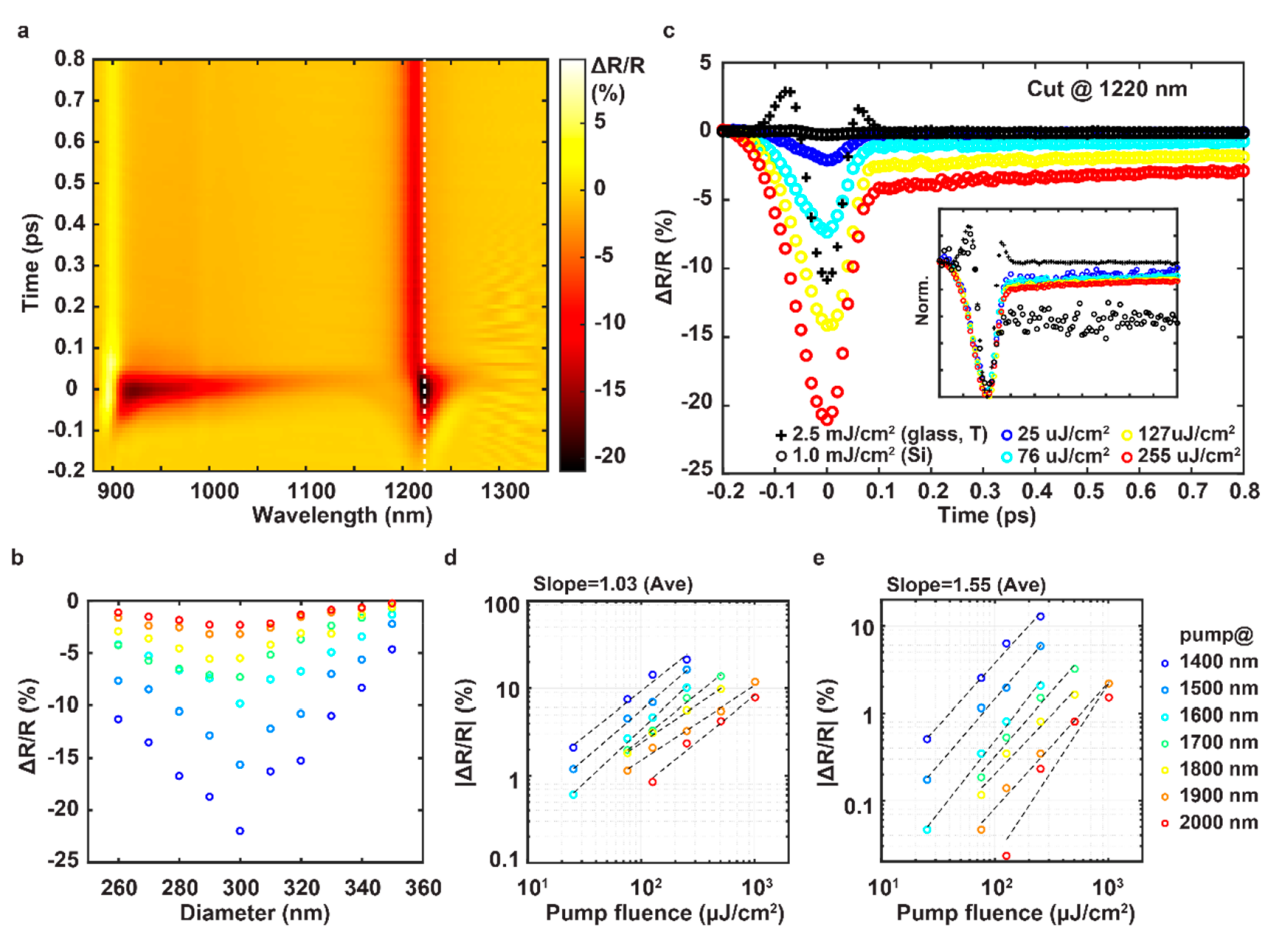
Transient optical response of the BIC metasurface. (a) Measured 2D map of reflective spectral change Δ*R*/*R* as a function of the probe wavelength and the time delay between the pump and probe pulses. The excitation wavelength is 1400 nm. (b) Maximum of Δ*R*/*R* on the MD resonance for varied pump wavelengths as a function of Si diameters at a fixed fluence of 255 μJ/cm^2^. (c) Metasurface pump fluence-dependent kinetics of Δ*R*/*R* at 1220 nm (white dashed line in panel a) compared to Δ*R*/*R* of the unpatterned Si film (black circles). The measurement from the glass sample (denoted as “+”) is transmittance change Δ*T*/*T*. The excitation is fixed at 1400 nm. The inset shows the normalized data. (d) Pump wavelength and fluence dependent |Δ*R*/*R*| due to the pulsewidth-limited ultrafast process measured at the time zero and the wavelength of 1220 nm for varied pump wavelengths. (e) Pump wavelength and fluence dependent |Δ*R*/*R*| due to the slow decay process measured at the time delay of 0.5 ps and the probe wavelength of 1210 nm for varied pump wavelengths. (b), (d), and (e) share the same legend shown in the inset of (e).

Additionally, there is no significant change in the local electric field, as the pump wavelength is highly detuned from the resonances for all experiments. The simulated electric field at different pump wavelengths does not vary significantly as shown in Supporting Information (Figure S7). Hence, the pump wavelength-dependent Δ*R*/*R* change is attributed to the dispersive nonlinear refractive index. We measure a Δ*R*/*R* of 22% for a sample with a nanodisk diameter of 300 nm and pump fluence of 255 μJ/cm^2^ which corresponds to a 0.9 pJ switching power per nanodisk. We calculate the switching speed using the plot of the Δ*R*/*R* kinetics at the probe wavelength of 1220 nm in Figure 2c and estimate a full-wave half-maximum (fwhm) of 100 fs for the ultrafast response. For comparison, the Δ*R*/*R* is only 0.1% at a 4-time larger fluence on the unpatterned deposited Si film. Thus, BIC modes supported by MD resonances enhance the signal modulation by a factor of 440. Pump power-dependent measurements change only the modulation amplitude and do not significantly affect the decay time. We estimate the IRF by measuring the transient transmittance response from a piece of 1 mm thick glass slide (Figure 2c, black “+”s). When compared to the ultrafast response, the IRF has very similar temporal behavior, demonstrating that the measured switching time is limited by the experimental setup. Thus, we estimate that the natural decay governing the ultrafast process is faster than the IRF by at least an order of magnitude, which coincides with the time scale of the bound electron dynamics.^27^ The modulation amplitudes for the pulsewidth-limited ultrafast process and the slow picosecond decay process are shown in Figure 2d and Figure 2e, respectively. The modulation amplitude of the ultrafast process is linearly proportional to the pump power, while the modulation amplitude of the slower decay is not linear to the pump power and the average fitted slope is between 1 and 2. The electron–phonon relaxation in gold films typically occurs on a picosecond time scale, and the nonlinear response is relatively weak,^40^ which deviates from both the measured ultrafast and slower decay. Therefore, we attribute the pulse-width-limited ultrafast decay to the third-order optical nonlinearity resulting from the nondegenerate two-photon transition process, with one pump and one probe photon occurring in Si. As for the slower decay, it can be attributed to a multiphoton absorption process of Si that involves two pump photons and contributions from a single photon absorption process due to the defects in amorphous Si. Both the two-photon and single-photon absorption processes can generate free carriers that exhibit relaxation and recombination dynamics that have picosecond time scales.

Next, we focus on the pulse-width-limited ultrafast process induced by the nondegenerate two-photon transition. This nonlinear transition corresponds to the imaginary part of the nonlinear refractive index when the sum of the pump and probe photon energies is above the band gap of the material. The resonance exhibits a shift and broadening feature that is reflected in the asymmetric shape of the transient spectra near the resonance of the metasurface. The two-photon transition involves both pump and probe photons, and thus, both the real and imaginary parts of the nonlinear refractive index should depend on both pump and probe wavelengths. We conducted two sets of pump–probe experiments varying each of two parameters *viz*. resonant wavelengths of metasurfaces and pump wavelength while holding the other constant (Figure 3a–h). The pulsewidth-limited response has a dispersive spectral shape with a prominent positive peak and a smaller negative dip. The positive peak increases more significantly with increased pump wavelength and diameter compared to the negative dip.

**Figure 3:**
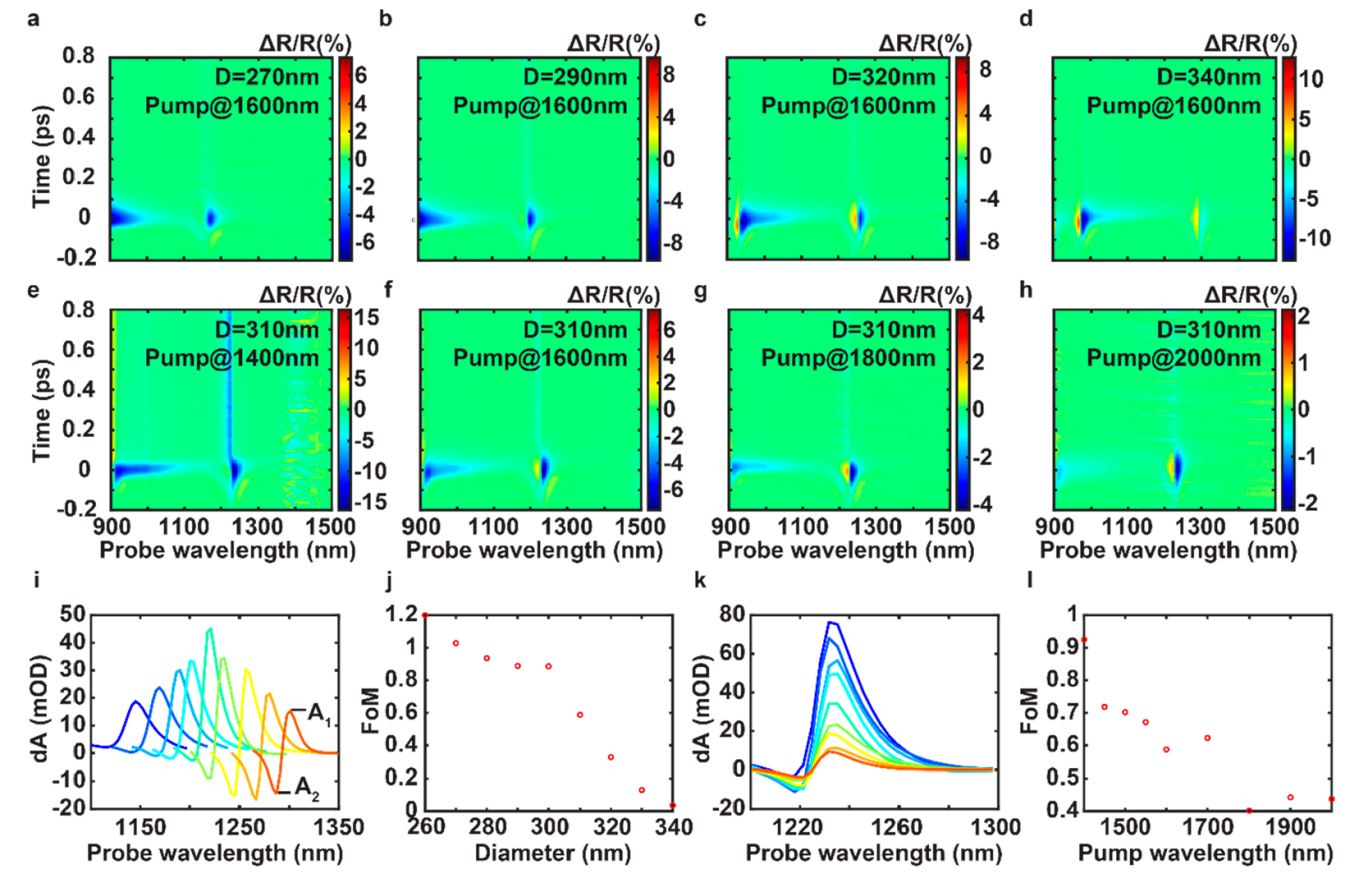
Evolution of ultrafast differential spectra with the BIC resonant wavelength and the pump wavelength. (a–d) Measured 2D maps of time-varied ΔR/R spectra of samples with different Si disk diameters pumped at a fixed wavelength. (e–h) Measured 2D maps of time-varying Δ*R*/*R* spectra of a selected sample with *D* = 310 nm pumped at different wavelengths. (i) Converted MD mode spectra d*A* spectra at time zero with different Si diameters from 260 to 340 nm represented by curves from blue to red. The pump wavelength is fixed at 1600 nm. The peak and dip values are denoted as *A*_1_ and *A*_2_, respectively. (j) Resonant wavelength (linked with the Si diameter) dependent *FoM* retrieved from (i), characterizing the spectral shape. (k) Converted MD mode d*A* spectra at time zero with varied pump wavelengths from 1400 to 2000 nm denoted by curves from blue to red. The Si pillar diameter is fixed at 310 nm. (l) Pump-wavelength-dependent *FoM* retrieved from (k).

Furthermore, we analyze the ultrafast transient spectra at zero time delay for different resonant and pump wavelengths. The difference in the reflectance spectra with (*R*_2_) and without (*R*_1_) the pump is calculated by converting Δ*R*/*R* into the change of effective optical density (OD) using d*A* = −[log_10_(*R*_2_) – log_10_(*R*_1_)] = −log_10_(1 + Δ*R*/*R*). Figure 3i shows the converted MD mode data at a zero time delay for metasurfaces with different diameters. All of the d*A* curves have a dispersive shape changing from negative to positive with the increased probe wavelength. The maximum positive and minimum negative values are denoted by *A*_1_ and *A*_2_, respectively. If we assume that the BIC resonance experiences a spectral shift without broadening, its transient response curve is expected to have an antisymmetric shape with |*A*_1_| = |*A*_2_|, i.e., *A*_1_ + *A*_2_ = 0. However, the measured d*A* curves do not follow an ideal antisymmetric shape, because of the resonance broadening. This broadening effect is analyzed using a normalized figure of merit (*FoM*) = (*A*_1_ + *A*_2_)/(*A*_1_ – *A*_2_) and is plotted as a function of the disk diameter in Figure 3j. We repeat the same procedure for varying pump wavelengths in Figure 3k. The corresponding *FoM*s as a function of the pump wavelength are shown in Figure 3l. A similar trend is also experimentally observed on the ED resonance (see Supporting Information, Figure S8). We observe that the *FoM* decreases with the increased diameter, which is related to the increased probe resonant wavelength. It also decreases with the increased pump wavelength, which may be caused by the dispersive imaginary part of the nonlinear refractive index induced by the TPA process. The dependence of *FoM* on the pump and resonant wavelengths is evaluated by modeling the dispersion of the nonlinear refractive index caused by the bound-electron third-order optical nonlinearity.

We use the two-band model to calculate the bound-electron nonlinear refractive index (Figure 4a inset). We calculate the nonlinear absorption coefficient that is directly linked to the imaginary part of the nonlinear refractive index and then obtain the real part using the K–K transformation. The nondegenerate nonlinear absorption process is dominated by TPA, Raman transition, and linear and quadratic Stark effects. The imaginary part of the nonlinear refractive index is given by
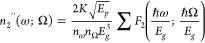
1where *K* = 3100, *E*_*p*_ = 21 eV, *E*_*g*_ is the band gap energy, and *n*_*ω*_ and *n*_Ω_ are the linear refractive indices at the probe and pump frequencies ω and Ω, respectively. Here we use *E*_*g*_ = 1.7 eV for amorphous silicon determined from the cutoff edge of the imaginary part of the linear index measured using ellipsometry (data shown in Supporting Information, Figure S9). Both *n*_*ω*_ and *n*_Ω_ are set as 3.5, and linear dispersion is neglected for simplicity. The function *F*_2_ has different forms when the various contributions mentioned above are considered,^31^ as shown in Supporting Information (eq S1). The K–K integral shown in eq 2 then gives the real part of the nonlinear refractive index.
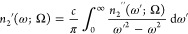
2The total pump intensity-dependent refractive index is defined as *n* = *n*_1_ + (*n*_2_′ + *n*_2_′′)*I*, where *n*_1_ is the linear index without the pump. The K–K relation can be used in this case, as the third-order complex susceptibility χ^(3)^(ω; ω, Ω, −Ω) obeys the K–K relation. Note that the K–K relation cannot be generalized to arbitrary nonlinear optical processes.^14,41^

**Figure 4:**
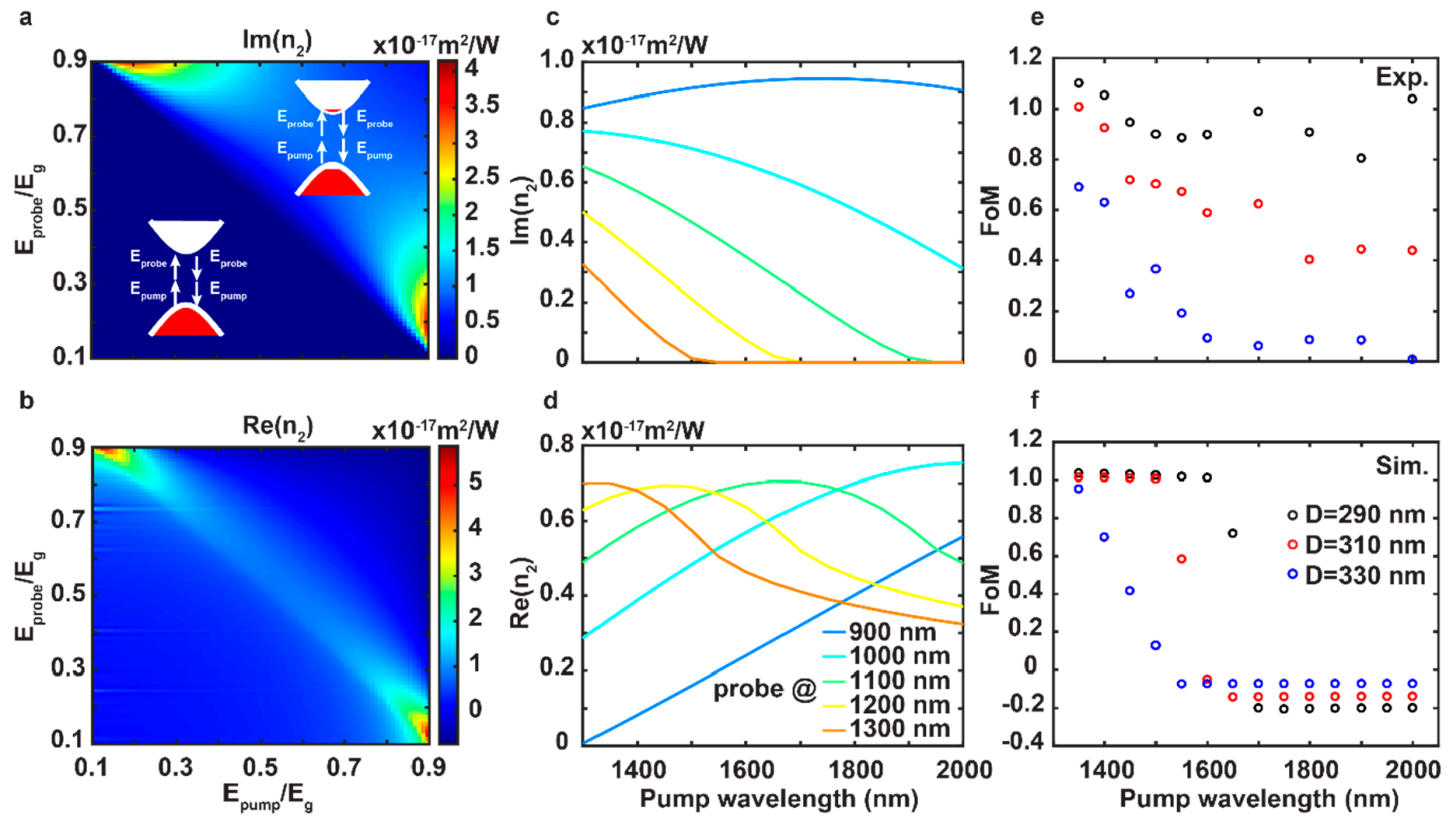
Dispersive nonlinear refractive index. (a, b) Calculated 2D map of the imaginary and real parts of the nonlinear refractive index as a function of the pump and probe photon energies. The insets show the energy diagram of the TPA process involving both pump and probe photons, which affects the complex nonlinear refractive index via the third-order optical nonlinearity of the material. (c, d) Calculated pump wavelength-dependent imaginary and real parts of the nonlinear refractive index with varied probe wavelengths from 900 to 1300 nm denoted by curves from blue to red, respectively. (e) Retrieved *FoM* from the measured spectra of different Si diameters with the fixed pump fluence of 255 μJ/cm^2^. (f) Retrieved *FoM* from the simulated spectra of different Si diameters using the calculated nonlinear refractive index, where the used pump intensity is the same as in experiments.

The calculated imaginary and real parts of the dispersive complex nonlinear index of Si are shown in Figure 4a and 4b, respectively. When the sum of the pump and probe photon energies is larger than the band gap energy, i.e. *E*_pump_/*E*_g_+*E*_probe_/*E*_g_ > 1, the probe photon is absorbed with the assistance of the pump photon. Otherwise, no absorption occurs, which corresponds to a zero value for the imaginary part and a nonzero value for the real part of the nonlinear index. This is equivalent to a four-wave mixing process described by χ^(3)^(ω = Ω + ω – Ω). The nonlinear indices are plotted as a function of pump wavelength for different probe wavelengths (900–1300 nm) in Figure 4c and 4d. The imaginary part decreases as pump and probe wavelengths increase, similar to the *FoM* in Figure 3j and 3l. Using the calculated nonlinear refractive index, we simulated the modulation of the metasurface and showed a significantly enhanced performance for the high Q quasi-BIC system compared with a low-Q MD mode (Supporting Information, Figure S10). We can also further analyze *FoM*s from the simulation results. The experimental *FoM*s from the measured spectra and the simulated *FoM*s based on nonlinear index calculations agree well with each other (Figure 4e–f). The few discrepancies can be attributed to the simplification used in the model for the nonlinear index which assumes only a direct band gap material with two bands. Amorphous silicon used in this study has no well-defined band structure with many defect states in the band gap which would need a detailed description of additional nonlinear absorptive processes and would be beyond the scope of this work. The model presented in the paper explains the fundamental features of the nonlinear transient processes observed in the experimental data. We believe that wavelength-tunable narrowband resonances of BIC metasurfaces make them good platforms for quantifying the dispersive complex nonlinear refractive index of materials.

In summary, we demonstrate an ultrafast and low-power all-optical switching platform using a BIC metasurface based on Mie resonances coupled to a mirror. The measured switching speed is on a femtosecond scale, limited by the laser pulse duration. The reflectance change of 22% is achieved at a fluence of 255 μJ/cm^2^ which is equivalent to 0.9 pJ per nanodisk and provides an enhancement by a factor of 440 when compared to an unstructured silicon film. This increase in performance can be attributed to the deep and high-Q resonance of BIC modes that enhance the optical nonlinearity of the bound electrons in the material. We analyze the broadening of the resonance using ultrafast differential spectra to understand the nature of the transient nonlinearity and ascribe it to the dispersive imaginary part of the complex nonlinear refractive index caused by TPA. A theoretical two-band model is used to calculate the complex nonlinear index and simulate the differential spectra, which show good agreement with experiments. These platforms and phenomena will enable high-speed optical switching devices and all-optical nonlinear chips for processing. Finally, our results indicate that BIC metasurfaces may be useful platforms for the characterization of dispersive optical nonlinearities using broad-band pump–probe techniques.

## Supporting Information Available

Materials and methods, details on nondegenerate absorption function, ground–ground state reflectance spectra, spacer thickness-dependent reflectance spectra, diameter-dependent reflectance spectra, details on the pump–probe measurement setup, simulated electric field distribution at different pump incident angles, simulated incident angle dependent reflectance spectra, simulated electric field distribution at different off-resonant pump wavelengths, ultrafast response of ED modes, measured refractive index of deposited silicon, numerical comparison of the modulation performance of Si disks on glass and on the gold film. (PDF)
