# Supplementary material for: Unlocking Efficient Ultrafast Bound-Electron Optical Nonlinearities via Mirror Induced Quasi Bound States in the Continuum
Source: Nano Lett. 2024 Jan 23;24(5):1679–86. doi: 10.1021/acs.nanolett.3c04431 (PMC10853962; doi:10.1021/acs.nanolett.3c04431)
Supplement: Supplementary file 1 — nl3c04431_si_001.pdf [file nl3c04431_si_001.pdf]

Supporting Information

Unlocking efficient ultrafast bound-electron optical nonlinearities via mirror induced quasi bound states in the continuum

Guoce Yang,[1] *Monica S. Allen,*[2] *Jeffery W. Allen,*[2] *and Hayk Harutyunyan,*[1,*]

[1]Department of Physics, Emory University, Atlanta, GA 30322, USA

[2]Air Force Research Laboratory, Munitions Directorate, Eglin AFB, FL 32542, USA

*hayk.harutyunyan@emory.edu

Materials and Methods

Numerical simulations

Electric field distributions were simulated using COMSOL Multiphysics. Ground-state spectra with varied spacer thicknesses and diameters were simulated by FDTD Solutions. The refractive index and thickness of Si and SiO_2 as well as the diameter of Si pillars used in the ground-state simulations were measured by ellipsometer and SEM. We use Johnson and Christy's gold refractive index value in simulations [1]. Differential reflectance spectra were obtained by calculating the spectra with and without the nonlinear refractive index contribution, and the spectra were simulated using rigorous coupled wave analysis (RCWA) programmed with the open-source MATLAB package [2].

Sample fabrication:

A 100 nm gold film with a 5 nm Cr adhesion layer is deposited on a silicon wafer substrate by a thermal evaporator. Next, SiO_2 and Si film are successively deposited on the gold film using plasma enhanced chemical vapor deposition (PECVD, Unaxis). To deposit SiO_2 film, we use 400 sccm SiH_4 (5% in He) and 900 sccm N_2O and process with a pressure of 900 mT, a power of 25 W, and a temperature of 250 °C. To deposit amorphous Si film, we use 1000 sccm SiH_4 (5% in He) and process with a pressure of 900 mT, a power of 50 W, and a temperature of 250 °C. Next, circle arrays are patterned on the Si film with the standard electron beam lithography (EBL) process. In the EBL process, PMMA A4 was first spun coated with the speed of 3000 rpm/s followed by 3 min baking at 150 °C, then exposed to electron beam with the area dose of 350 μC/cm2, and finally developed in MIBK: IPA=1:3. The 60 nm thick Al film was evaporated on the sample by thermal evaporation at the pressure below 2×10^{-7} Torr, and lifted off after the immersion in acetone overnight. The silicon without the covered Al mask was vertically etched by inductively coupled plasma reactive ion etching (ICP-RIE, Plasma Therm) with 50 sccm Cl_2, a platen power of 125 W, a coil power of 75 W, and a pressure of 5 mT. After removing the remaining Al mask layer with Al etchant, the final metasurfaces sample was achieved.

Optical measurements

The ground-state infrared reflectance spectra of the metasurfaces are acquired by a microscope and grating spectrometer with an InGaAs line array detector. The white light is guided from an incandescent lamp source to an objective with a numerical aperture (NA) of 0.25, and a pinhole is placed at the back aperture of the objective to further reduce the NA to ~0.03. The reflected white light from the metasurface positioned on the focal plane is collected by the same objective

and guided to the input slit of the spectrometer. The transient reflectance differential spectra are acquired with a pump-probe optical setup. A 5 mJ amplified Ti: Sapphire system (Coherent Astrella) with a pulse duration time of 35 fs and a repetition rate of 1 kHz working at the central wavelength of 800 nm is used as the primary pump source. The output beam is divided into two paths. One is sent to the optical parametric amplifier (OPA) to generate a wavelength-tunable pulse used as a pump beam. The other is sent to a delay line and then focused on a crystal to generate a broadband white light pulse used as a probe beam. The incident angle of the pump and probe beams on the metasurfaces are ~ 30° and 5°, respectively. Both polarizations are s-polarized. The reflected probe beam is coupled to a fiber linked to a spectrometer which has a wavelength resolution of 3-4 nm in the infrared region. A chopper working at 500 Hz is used to modulate the pump beam so that the ratio of reflected intensities with and without the pump is recorded.

Equation (S1)

Nondegenerate absorption function $F_2(x_1;x_2)$

$$F_2(x_1;x_2)=\begin{cases}\dfrac{\theta(x_1+x_2-1)^{3/2}}{2^7x_1x_2^2}\left(\dfrac{1}{x_1}+\dfrac{1}{x_2}\right)^2 & \text{(A)}\\ \dfrac{\theta(x_1-x_2-1)^{3/2}}{2^7x_1x_2^2}\left(\dfrac{1}{x_1}-\dfrac{1}{x_2}\right)^2 & \text{(B)}\\ -\dfrac{\theta(x_1-1)^{3/2}}{2^6x_1x_2^2}\cdot\dfrac{1}{x_2^2} & \text{(C)}\\ -\dfrac{1}{2^{10}x_1x_2^2\theta(x_1-1)^{1/2}}\left(\dfrac{1}{x_1-x_2}-\dfrac{1}{x_1+x_2}\right) & \text{(D)}\end{cases} \qquad \text{(S1)}$$

$F_2(x_1;x_2)$ shown in Eq. (1) in the main text has different forms depending on the nonlinear contributions. Here we list the specific forms we used in our calculations of the nonlinear refractive index. In Eq. (S1), $\theta(x)=x$ only when $x>0$, otherwise $\theta(x)=0$. (A), (B), (C) and (D) represent the contribution from two-photon absorption, Raman transition, linear Stark effect and quadratic Stark effect, respectively.

Figure S1

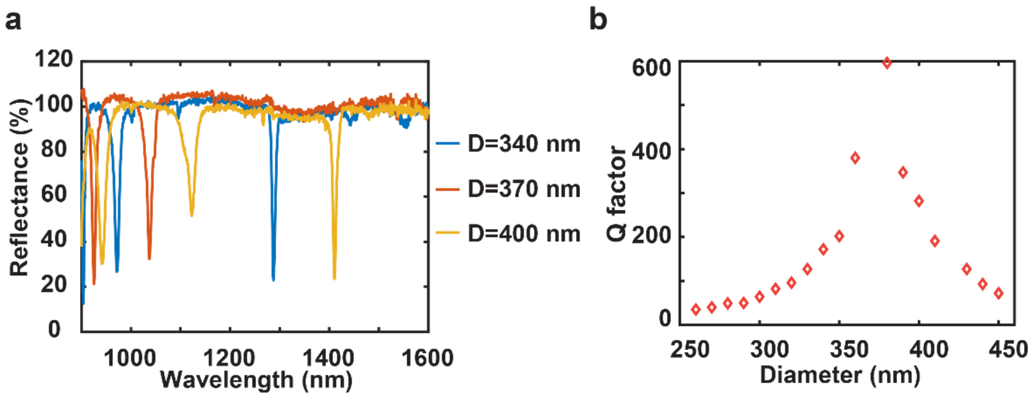

Figure S1. (a) Representative measured reflectance spectra of metasurfaces with diameters of 340 nm, 370 nm and 400 nm. (b) Retrieved Q factors as a function of the pillar diameter in experiments.

Figure S2

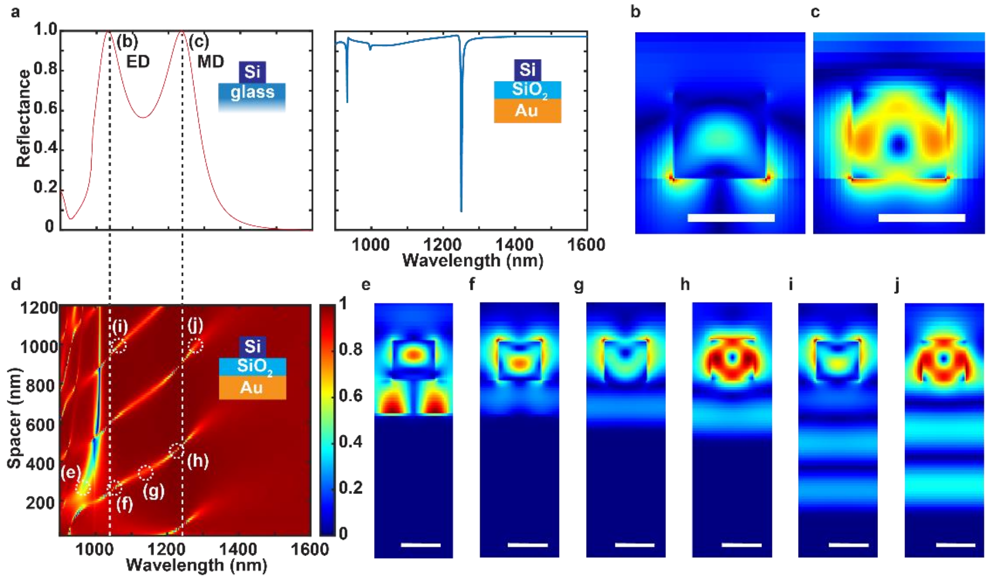

Figure S2. Spacer thickness-dependent reflectance spectra. (a) Simulated reflectance spectrum of Si disk array on glass vs on a gold mirror (the diameter is 330 nm and the period is 660 nm) showing ED and MD resonances. The spacer thickness is 80 nm in the case of Si disks on a gold mirror. (b, c) Simulated electric field profiles of ED and MD modes marked in (a), respectively. Scale bars: 300 nm. (d) Simulated reflectance spectra of Si disk array with the same geometry as (a) on the gold mirror as a function of spacer thickness varying from 20 nm to 1200 nm. With the increased spacer thickness, both ED and MD BIC turning points appear repeatedly and the resonances match well with the Si disk array. (e-j) Simulated electric field profiles corresponding to different positions in (d). Scale bars: 300 nm. Specifically, (e) shows the coupling between ED mode and surface plasmon polaritons (SPPs) mode, which could impact near field coupling rate and the effective refractive index of the medium between the center of the Si disk and the gold surface. (f-h) shows the evolution from the ED mode to the MD mode with the different spacer

thicknesses of 260 nm, 350 nm and 450 nm. (i) and (j) show the ED and MD modes, which keep well with the even thicker spacer (1000 nm).

Figure S3

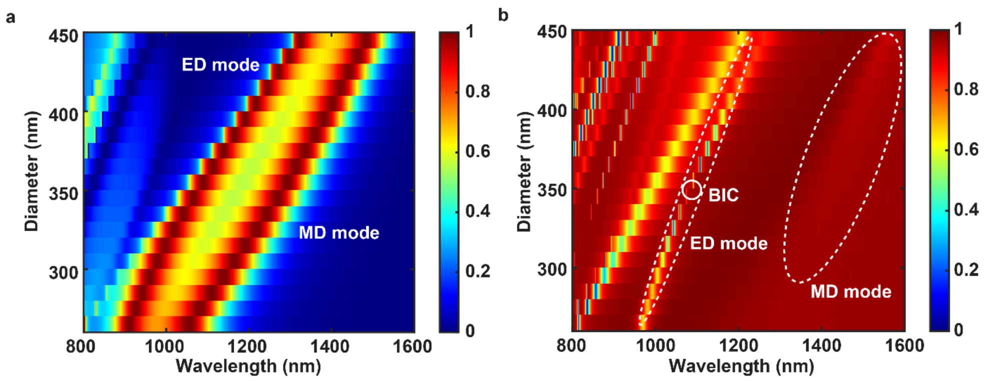

Figure S3. Diameter-dependent reflectance spectra. Reflectance of Si array on glass (a) and the gold mirror separated by 260 nm SiO2 (b) as a function of wavelength and diameter. The period is the double of the diameter. In (b), MD mode is not as clear as ED mode because the MD mode is far away from the BIC turning point at this specific spacer thickness (see Fig. S2 d).

Figure S4

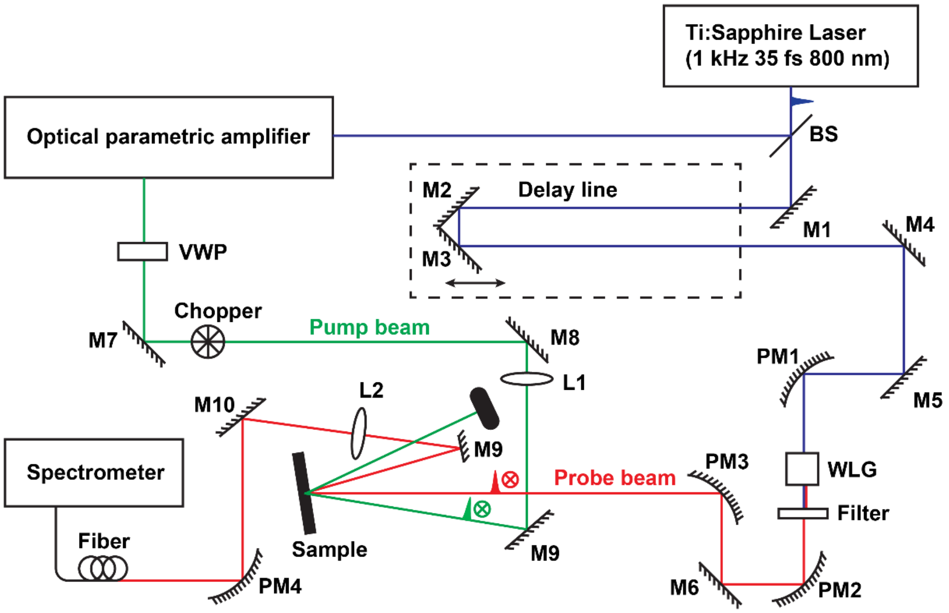

Figure S4. Pump-probe measurement setup. Schematic of pump-probe experimental setup. Blue lines represent the primary pump beam from the amplified Ti: Sapphire laser. Green lines represent the wavelength-tunable light from the Optical parameter amplifier (OPA) output. Red lines represent the broadband white light as the probe beam. M1-M10 are planar mirrors, PM1-PM4 are parabolic mirrors, L1 and L2 are lenses, BS is a beam splitter, WLG is a white light generator, and VWP is a variable wave plate. The purpose of inserting a VWP is to change the linear polarization angle of the pump beam. The incident angle of the pump and probe beam on the measured sample is ~5° and ~ 30°, respectively. Both incident beams are s-polarized.

Figure S5

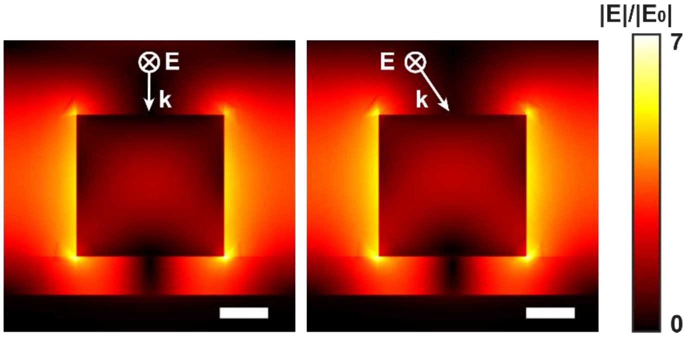

Figure S5. Simulated electric field distribution at the pump wavelength of 1400 nm with different incident angles of 0° and 30°. Scale bar: 100 nm.

Figure S6

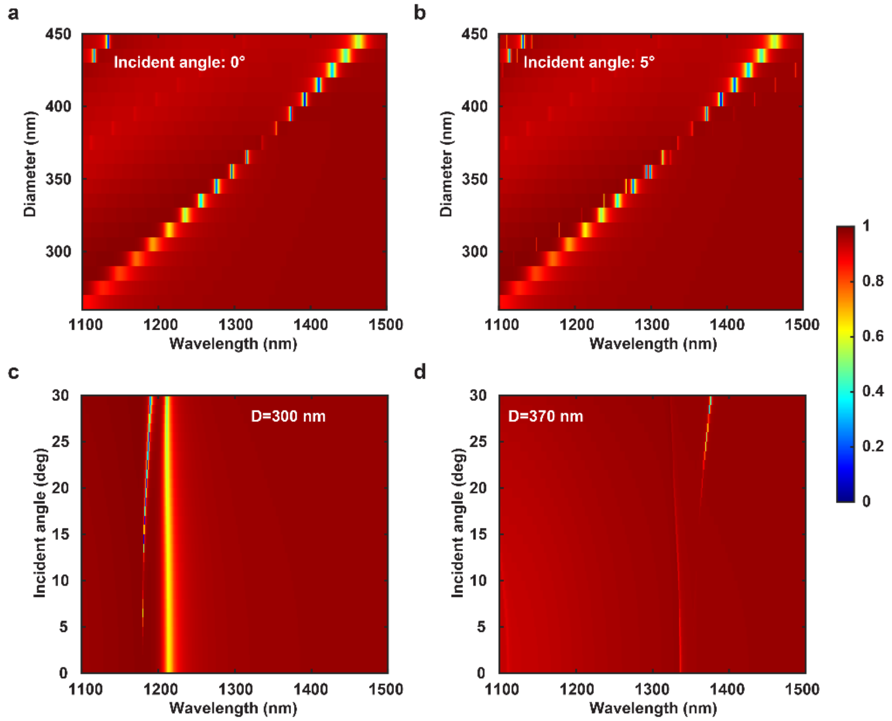

Figure S6. Simulated incident angle dependent reflectance spectra. (a) and (b) show the map of reflectance as a function of the wavelength and the diameter of the nanopillars at the normal incident case (incident angle of 0°) and at the incident angle of 5°, respectively. (c) and (d) show the map of the reflectance as a function of the wavelength and the incident angle at D=300 nm and D=370 nm, respectively.

Figure S7

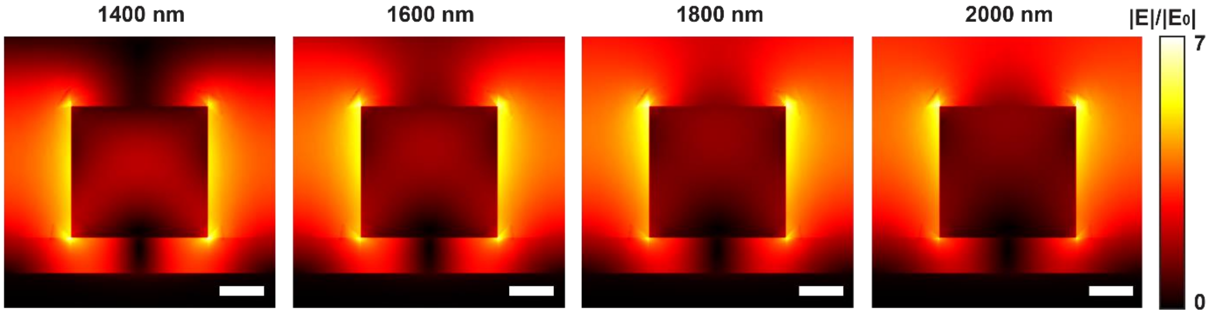

Figure S7. Simulated electric field distribution at different off-resonant pump wavelengths for a Si pillar resonant at 1210 nm. Scale bar: 100 nm.

Figure S8

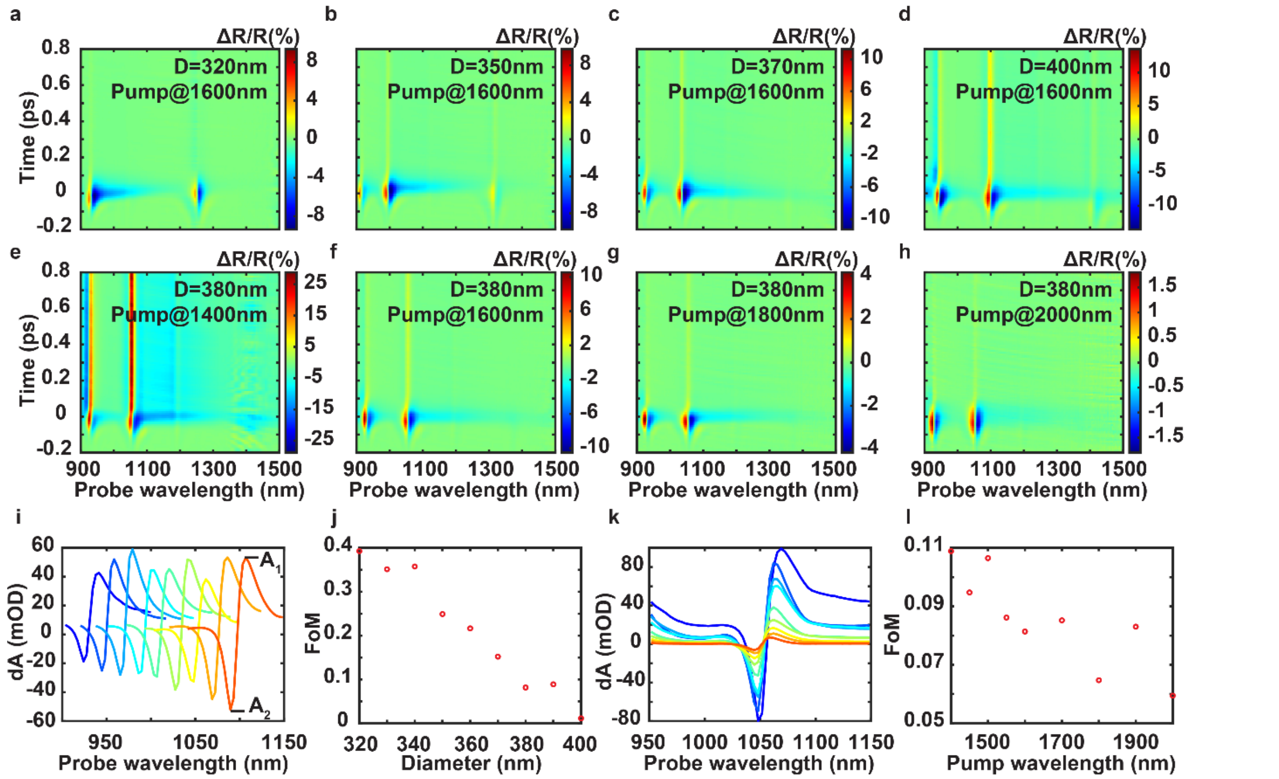

Figure S8. Measured diameter and pump wavelength-dependent ultrafast differential spectra. (a-d) Measured 2D map of time varied Δ*R*/*R* spectra of samples with different Si diameters pumped at a fixed wavelength. (e-h) Measured 2D map of time-varying Δ*R*/*R* spectra from the same sample but pumped at different wavelengths. (i) Converted dA spectra at time zero with different Si diameters from 320 nm to 400 nm represented by curves from blue to red. The pump wavelength is 1600 nm. Denote the peak and dip values as A_1 and A_2. (j) Resonant wavelength (linked with the Si diameter) dependent *FoM* retrieved from (i), characterizing the spectral shape. (k) Converted d*A* spectra at time zero with varied pump wavelengths from 1400 nm to 2000 nm denoted by curves from blue to red. The Si diameter is 380 nm. (l) Pump wavelength-dependent *FoM* retrieved from (k).

Figure S9

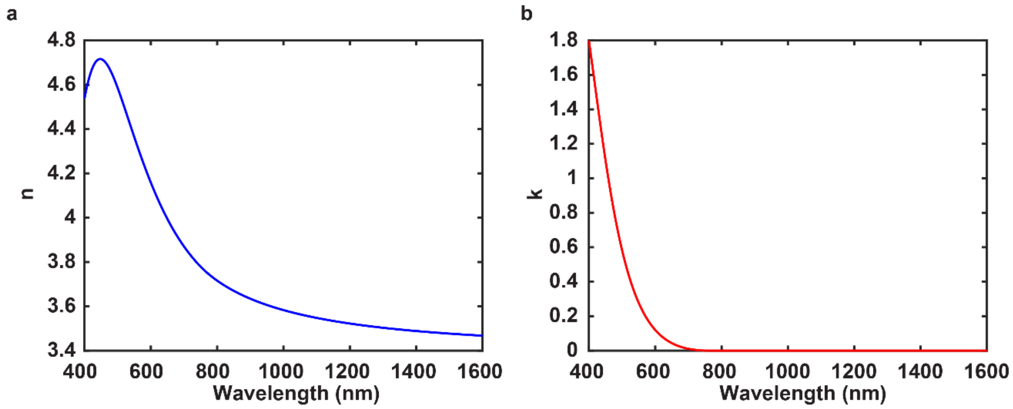

Figure S9. Measured refractive index of deposited silicon. The measured complex linear refractive index of deposited amorphous silicon. (a) Real part. (b) Imaginary part. The cut-off edge of the imaginary part is 728 nm.

Figure S10

To demonstrate how the high Q factor can benefit the switching performance more directly, we simulated ΔR of Si nanopillars on glass (low Q factors) and compared it with the results of Si nanopillars on gold (high Q factors due to BIC). Here, the MD resonant depths and wavelengths of the two cases are nearly the same. The simulation results are shown in Figure S10 below. We can see that the higher Q factor does enhance the modulation by two orders of magnitude.

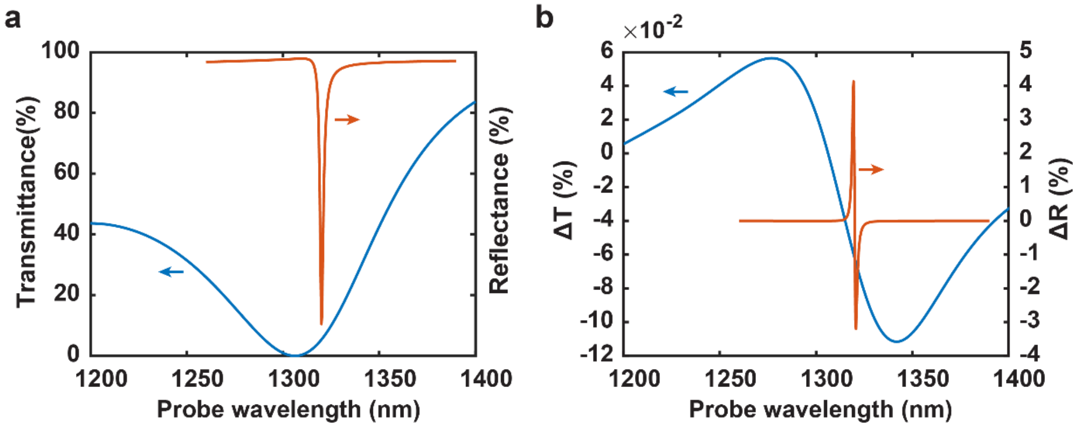

Figure S10. (a) Simulated ground-state spectra of Si pillar array on glass (blue line) and on Au mirror (red line). The size of the Si pillar in both simulations is 350 nm. (b) Simulated pump pulse-induced spectral modulation when pumped at 2000 nm with the fluence of 255 μJ/cm^2.

References

1. Johnson, P. B. & Christy, R. W. Optical constants of the noble metals. Physical Review B 6, 4370 (1972).

2. Hugonin, J. P. & Lalanne, P. Reticolo software for grating analysis. arXiv preprint arXiv:2101.00901 (2021).
